# The use of system dynamics for energy and environmental education

**DOI:** 10.1186/s41239-021-00309-3

**Published:** 2022-01-28

**Authors:** Alexandre Strapasson, Marcello Ferreira, Diego Cruz-Cano, Jeremy Woods, Marco Paulo do Nascimento Maia Soares, Olavo Leopoldino da Silva Filho

**Affiliations:** 1 Belfer Center for Science and International Affairs, Harvard Kennedy School, Harvard University, 79 JFK Street, Cambridge, MA 02138 USA; 2 Centre for Environmental Policy, Imperial College London, London, UK; 3 Institute of Physics, University of Brasilia (UnB), Brasilia, Brazil; 4 College of Engineering, University of Texas at El Paso (UTEP), El Paso, USA

**Keywords:** System dynamics, 2050 Calculator, Environmental education, Science teaching, Critical pedagogy, Climate change, Paulo Freire

## Abstract

The use of system dynamics as a learning tool for developing sustainable energy strategies and environmental education has advanced in recent years with the availability of new modelling software and webtools. Among the existing models, we highlight the online 2050 Calculators, which aim at simulating scenarios for greenhouse gas emissions, energy planning, sustainable land use, and food consumption. The objective of this study is to assess the available calculators and their contribution to an interdisciplinary education via systems thinking. We carried out a review of the existing models worldwide and ran some of the tools with students from three different postgraduate programmes at master’s level at Imperial College London (United Kingdom) and IFP School (France), whilst also assessing their individual views afterwards. The assessments were conducted once a year during three subsequent years: 2019, 2020, and 2021. The results are discussed under the epistemology of critical pedagogy, showing that the use of webtools, such as the 2050 Calculators, can significantly contribute to the students’ environmental awareness and political engagement, providing important lessons about the use of system dynamics for policy and science education.

## Introduction

System dynamics is a modelling approach based on the variation of stocks and flows over time. It can be used, for example, for modelling the dynamics of oil reserves and the impacts of oil exploitation on climate change over the years as well as the potential of bioenergy production in the coming decades against food security and sustainable land use, among many other possible examples. These dynamics can be integrated with several other variables, forming a complex system model, which can be applied for different areas, from social sciences to physics and maths. One of the main patrons of its use in science education was late Professor Jay Forrester from the Massachusetts Institute of Technology (MIT) in the 1960s and 1970s (Forester, 1992; Strapasson, 2014). Since then, system dynamics has become largely used worldwide, particularly after the publication of “The Limits to Growth” by the Club of Rome in the 1970s (Meadows et al., 1972), which used system dynamics for simulating global development scenarios and projecting potential environmental impacts over time. Several modelling software focused on system dynamics (Voinov, 2008) evolved from these experiences, such as *Stella*, *Vensim*, *Powersim*, as well as some visual tools for systems thinking, such as *Loopy* and *iThink*; however, it is also possible to develop system dynamics models using more general software, such as *MS Excel*, *Matematica*, *Visual Basic*, *R*, *Ruby, Knime*, *Phyton*, *C*, and other languages. The use of system dynamics to assess planetary boundaries became largely adopted in many research areas (Sverdrup & Ragnarsdottir, 2011).

In this context, the United Kingdom’s Government published in 2010 a whole-system model for carbon mitigation pathways by 2050, integrating several energy variables into a single operational online model, called the “UK 2050 Calculator” (Amakpah et al., 2016). This calculator was motivated by late Professor David MacKay from the University of Cambridge (Kiso, 2020), whilst Chief-Scientist at the former UK Department of Energy and Climate Change—DECC (currently UK Department for Business, Energy & Industrial Strategy—BEIS), based on the principles of his book “Sustainable Energy: Without the Hot Air” (MacKay, 2008). Since then, several other nations have developed their own calculators (Appendix 1), such as, Australia, Austria, Belgium, Bangladesh, Brazil, China, Colombia, India, Indonesia, Japan, Mauritius, Mexico, New Zealand, Nigeria, South Africa, Southeast Europe, South Korea, Switzerland, Taiwan, Thailand, United States, and Vietnam, all of them influenced by the UK version, which was recently updated.^1^ The 2050 Calculators have been used by policy makers, business leaders and NGOs for discussing carbon mitigation pathways. The calculator is a science-based model, made available online and in open access, with transparency and a relatively simple web interface. They have been used in several places, from universities and secondary schools to governments, companies, and international conferences.

In addition to the national calculators, a group of international researchers published the Global Calculator^2^ (GC), which was launched at the UK Royal Society in 2015. The GC was a pioneering initiative to model greenhouse gas emissions, energy, and land use change by 2050. It is a single integrated model designed at global scale, based on system dynamics. The project was led by the former UK DECC and Climate-KIC (European Union), involving several partners, such as Imperial College London, World Resources Institute (WRI Washington), Climact (Belgium), E&Y (India), PIK-Potsdam (Germany), the China’s Energy Research Institute (ERI), and the London School of Economics (LSE). Soon afterwards, the Financial Times also published its own climate change calculator,^3^ developed by Imperial College and the Indian Institute of Science (IISc Bangalore), for simulating carbon mitigation scenarios according to the Paris Agreement of the United Nations Framework Convention on Climate Change (UNFCCC, 2015). Moreover, the European Union has supported the development of the European Calculator (EUCalc),^4^ through its Horizon 2020 Programme. The calculators can also be deployed at city level, such as the Beijing 2050 Calculator and the Wellington 2050 Calculator, as well as for companies, such as the Bulb Calculator^5^ (Strapasson et al., 2020a). Therefore, these types of system dynamics models have been proven successful communication tools, with several initiatives worldwide already implemented and others in process.

The system dynamics of complex issues, such as energy, land use dynamics and climate change mitigation strategies, can provide a broad and critical understanding of sustainable development issues. The use of systems science (which includes system dynamics models) has been adopted for different purposes, from natural resources’ dynamics and engineering problems to social interactions and linguistics (Korn, 2009). Some scholars have already assessed the use of modelling tools for teaching science courses such as Physics (Brandão, 2008; Macêdo et al., 2012; Mozena & Ostermann, 2016) as well as the use of graphing calculators with teenage students for the development of graphical concepts with technology (Yaakub, 2002). The use of system dynamics in science education is not new (Chow & Cheung, 1992; Forester, 1992; Gould-Kreutzer, 1993), but the available studies have not covered yet the use of virtual system dynamics models for energy and environmental education, such as the 2050 Calculators. Moreover, there is also a lack of practical case-studies about their use at classroom level (in person and by distance mode). This gap of knowledge motivated the development of this study, particularly due to the importance of these tools already available in several nations and the global climate emergency.

In addition, the pandemic of COVID-19 brought some new challenges for distance education. Several schools worldwide intend to either keep or expand the use of digital environments, whilst others may change to hybrid learning (combining physical with virtual activities), rather than returning to fulltime face-to-face mode exclusively. The fact that the calculators are available online and in open access represents a significant advantage for virtual education. It is worth noting that the calculators are just an example of a system dynamics tool that can be used for education, given that there are several other digital tools that are equally important and aimed at different ends, such as for biodiversity conservation, pollution control, water use, mining, sustainable lifestyles, ecology, and cultural heritage (Liritzis & Volonakis, 2021). The models should be chosen according to their purposes, while also considering the need for critical thinking to test hypotheses (Tredennick et al., 2021). Whenever appropriate, they should be combined with experiential activities for a better effective learning, such as technical visits, field work, community work, and other extra-classroom activities.

Therefore, more than just another didactic tool, the calculators can be used to empower the society to find science-based solutions for major sustainable development issues, such as climate-energy-food-water nexus, through systems thinking. The universities, for example, could use this type of models as an educational tool more often, including in undergraduate courses, such as Physics, Engineering, Environmental Science and Economics, and postgraduate programmes, offering to the students a critical pedagogy. Secondary Schools (High Schools) can also be benefited from these type of open access webtools. Some calculators, for example, also have a light version available (Appendix 1), like a videogame, which can be useful for teaching to young students, especially to complement discussions in science and geography about climate change mitigation pathways. On the other hand, the focus of this study was to assess its use in higher education.

## Objectives

The main objective of this study was to assess the effectiveness of using system dynamics models (2050 Calculators) for energy and environmental education, whilst also offering insights for educators interested in this type of pedagogical approach.

The specific objectives were to:

- Identify and compare current calculators in terms of their affordability as a didactic material;
- Assess the usefulness of these models with students in classroom environment (physically and virtually) in three different postgraduate programmes at master’s level, one at Imperial College London (United Kingdom) and two at IFP School (France), from 2019 to 2021;
- Discuss their contribution in the light of the critical pedagogy approach and other complementary epistemological references.
- Review the students’ suggestions on the use of system dynamics models in education through an anonymous survey.

## Literature review

This brief literature review focuses on two main issues, the 2050 calculators and the epistemological approach used for the teaching practice. This review is provided to support the methodology and to contextualise the results and discussions subsequently presented.

### Overview on the 2050 calculators

This study is focused on the educational aspect of the calculators and, therefore, only an overview on the technical part of these models is here shown. However, by accessing the references and weblinks provided in this article, it is possible to further explore and run these calculators online. A more in-depth discussion on how the calculator’s models work, and the main dynamics and uncertainties involved can be found in Strapasson et al. (2017, 2020b, 2020c). In addition, the Global Calculator, more specifically, has two short tutorial videos^6^^,^ 7 explaining its importance and how to use it for running simulations, which can be used for didactic purposes as well. Other calculators have guiding documentations to assist the user on how to use them, and policy briefs on sectorial assessments (Baudry et al., 2020; Costa, 2020; Warmuth et al., 2020; Vitali et al., 2020; Martin & Pestiaux, 2020; Gyalai-Korpos, 2020; Yu & Clora, 2020; Setter et al., 2020; Strapasson et al., 2020d). Most of the available calculators use 2050 as a target year, given that this is the reference date currently used in international negotiations at the United Nations, including the UNFCCC Paris Agreement.

As system dynamics models, the 2050 Calculators operate based on stocks and flows of natural resources (e.g. oil, gas, land, energy), which can vary overtime by linking supply and demand across all sectors of the economy, such as transport, power generation, food consumption, land use, industry (manufacturing), and buildings. Hence, all sectors are interconnected, involving several complex calculations. For instance, if the calculator’s user projects a scenario in which the population would eat more meat or consume more energy, the land and energy sectors, respectively, would have to be able to supply these projected demands and so forth. Some 2050 Calculators are focused on the energy sector only, while others also include crop and livestock yields, dietary patterns (e.g. plant-based food vs. meat), forest, biodiversity and water conservation. The challenge is to provide an integrated model with a relatively simple interface for a non-expert user and, at the same time, present a reliable and transparent tool, which is scientifically consistent and not politically biased.

Several studies addressed the use of calculators to assess different geographical and sectorial impacts on climate change (Berger et al., 2020; Cooper et al., 2016; Elizondo et al., 2017; Gyalai-Korpos et al., 2020; Moinuddin & Kuriyama, 2019; Roscini et al., 2020; Strapasson, 2016; Strapasson et al., 2017, 2020b), as well as the effects of lifestyle changes (Costa et al., 2021) and employability (Füllemann et al., 2019). Regardless of different sectorial approaches and alternative features of some models, all 2050 Calculators follow a similar logic, which builds on the pioneering initiative of the UK 2050 Calculator.

**Fig. 1:**
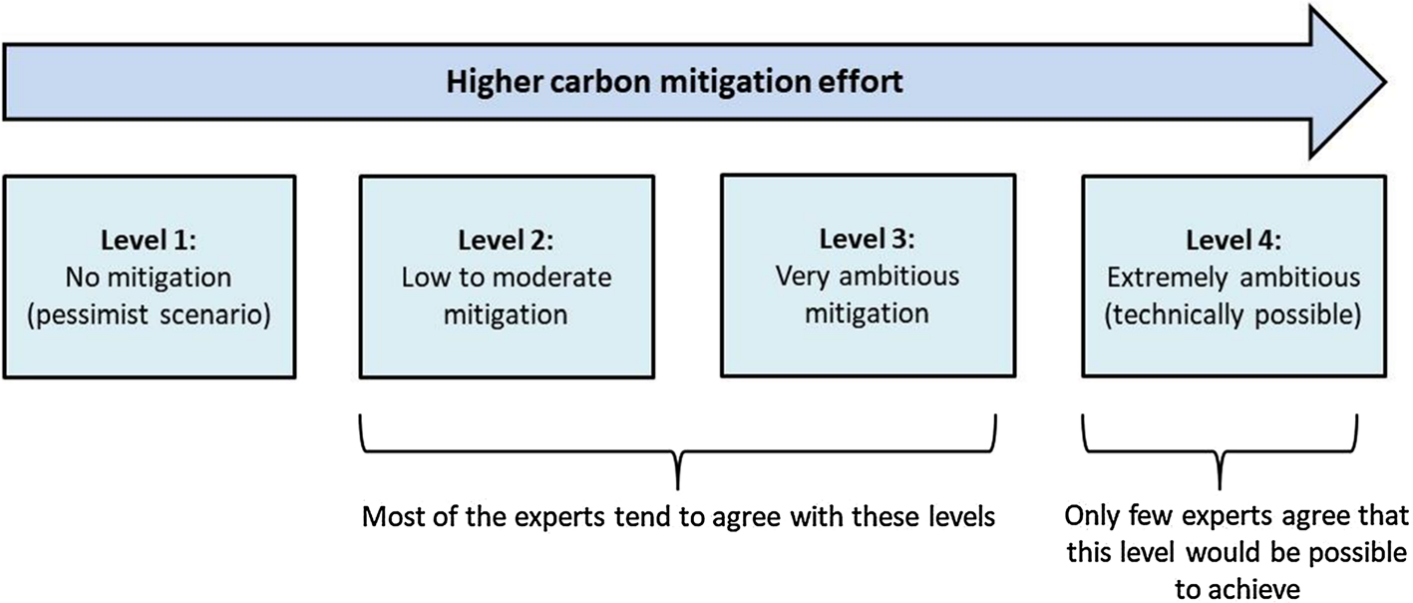
Calibration of lever’s levels in the 2050 Calculators. Source: prepared by the authors, adapted from the Global Calculator

The calculators work based on several representative “levers”. A lever is a vector which significantly affects greenhouse gas (GHG) emissions over time, either positively or negatively. Some examples of levers are the use of solar energy, nuclear power, oil and gas, bioenergy and carbon capture and storage (CCS) technologies, among many other possible vectors. As shown in Fig. 1, each lever has (in most calculators) four “levels” of growing ambition to carbon mitigation, from level 1 to level 4, all of them from the current year until 2050. Thus, the users can change the levers’ levels according to their development preferences in order to simulate different carbon mitigation pathways. It is worth noting that the calculators are engineering tools for running free simulations online to help inform and discuss sustainable policy strategies, rather than prediction models, such as *Markal*, *Times*, *Message*, *TIAM, GTAP, GLOBIOM*, and other approaches for scenario simulations (Vallejo et al., 2021), which are all equally important and aimed at different audiences and purposes.

All 2050 Calculators currently available were used and tested by the authors to verify, before using them in classroom, if they have affordable interfaces, transparency, and the possibility of being used for a critical and interdisciplinary education. All models can be checked using the links already provided to the respective models (Appendix 1). Thus, this experiment carried out in this study can be repeated by running the models directly with students from other universities or schools in different nations, languages, and cultures. However, the results may vary according to the characteristics of the chosen school, their entrance requirements, the social context, and previous knowledge of the students involved in the assessment, the lecturer’s teaching skills, among other aspects.

### Epistemological approach

The epistemological basis of this study is the “critical pedagogy”, which involves an interdisciplinary and radical understanding of the perspective of human emancipation. As a theoretical-practical current (of conscious action), critical pedagogy challenges social, political, economic, and cultural relations in their complex determinations and contradictions, seeking to problematize them and transform them into an educational sphere based on reflection, dialectics, and dialogism. From the school and university angle, the critical perspective can be applied through a curriculum that is effectively constituted as an identity document (Silva, 2010) and that selects, organizes, and mobilizes teaching, learning and evaluation practices. Thus, the teacher must consider the local, socioeconomic, and cultural conditions of the students, mobilizing conscious and autonomous reflections on development issues, which question and challenge business-as-usual patterns. In this context, system dynamics tools have an evident potential to critical pedagogy, especially because they can make use of knowledge and articulations that simulate contradictions of socioeconomic, technological, and environmental aspects. These tools can be used, for example, to inform sustainable development pathways for climate change mitigation, among several other applications. Their contents and devices can incorporate subsidies for critical reflection and the subversion of unsustainable development trends at local, national, and global scale.

The main reference of critical pedagogy used in this assessment is the Brazilian Philosopher Paulo Freire, who wrote about the role of a critical education in freeing up people from oppressive systems (Freire, 2005, 2011a, 2011b, 2011c). In the context of the calculators, the oppression is represented by negationist views about climate change (or that this problem should not be tackled rapidly), for example, from part of the mainstream oil and gas industry and governments. Freire suggested that people should look for a libertarian education, in contrast to a mainstream education, also called by him as a “banking education”, which is not concerned with preparing critical and autonomous citizens, but instead to maintain the global establishment, whilst also passively accepting social inequalities (Freire, 2011b, 2011c; Moreira, 2011). Thus, in such a pedagogic approach, the use of system dynamics models, among other tools, initiatives, and didactic materials, could potentially help society to autonomously obtain a critical systems-view on their problems. Hence, models such as the 2050 Calculators should be built by a multidisciplinary team and as a bottom-up initiative, i.e. led by local researchers rather than as a top-down initiative led by large corporations or international consulting firms, which, on the other hand, can contribute as modelling partners by sharing their experiences and knowledge.

Critical pedagogy presupposes a praxis (conscious action), a connection between educational practice, art, social, economic, and cultural justice, as well as the protection of human rights and democracy (Freire, 1979, 2000, 2005; Boal, 2013). The calculators can be seen as a relatively small (although important) contribution to stimulate critical thinking and systems view, particularly on climate change mitigation strategies, without policy and corporation biases, and that are accessible in open access to everybody with internet access, from public schools in rural areas of developing nations to elite institutions worldwide.

Another important related reference in critical pedagogy is Henry Giroux, who claims that social transformation and individual emancipation are always potential outcomes of this connection, whereas “education” is its activator (Giroux, 1997). As he stated in his own words “(…) critical educators provide theoretical arguments and large volumes of empirical evidence to suggest that schools are, in fact, agencies for social, economic and cultural reproduction. At best, public-school education offers limited individual mobility to members of the working class and other oppressed groups, but, ultimately, public schools are powerful instruments for the reproduction of capitalist relations of production and of legitimatising ideologies of everyday life” (Giroux, 1997, p. 148, translated by the authors). Therefore, in the context of the 2050 Calculators, the challenge is to move from mainstream education based on business-as-usual trends and markets to an education that can stimulate critical thinking about sustainable development pathways and behavioural changes.

In addition, some other psychological learning theories were considered during the teaching practices, particularly those from David Ausubel, Lev Vygotsky, and Philip Johnson-Laird, as further briefly addressed in “Results and discussion”. From Ausubel, we consider his *Meaningful Learning* theory (Moreira, 2011; Silva Filho & Ferreira, 2018), in which new contents must make sense to the students in the context of knowledge they already have. From Vygotsky, we use his concepts of learning through social interaction and the *Zone of Proximal Development* (a.k.a. *Zone of Imminent Development*), in which true learning can be more effectively achieved (Vygotsky, 1991, 2000). As for Johnson-Laird, we consider his ideas on *Mental Models* and human reasoning (Johnson-Laird, 1995, 2010).

## Methodology

This research was conducted by assessing the 2050 Calculators currently available online and in open access. All the three lectures involved in this assessment were about an introduction to renewable energies with an emphasis on bioenergy, and carbon mitigation strategies (Pacini & Strapasson 2012; Smith et al., 2014; Strapasson et al., 2019, 2020b). The calculators were used as an interactive systems model for participatory discussion with the students in the context of the global climate debate. The models are aligned with the UNFCCC’s Paris Agreement and the United Nations Sustainable Development Goals (SDGs),^8^ with an emphasis on goals no. 7 (“affordable and clean energy”) and no. 13 (“climate action”). The first part of these lectures was more technical (overview on renewable energy technologies and concepts). In the second part, the calculators were demonstrated along with the learners in classroom (either in person or online). After this, their personal views and impressions were anonymously assessed through a survey. The following sections provide a description on the calculator’s approach and how they were used and assessed with the students.

### Description of the sample groups and timeline

The main characteristics of the three different postgraduate programmes (master’s level) in which the 2050 Calculators (specially the Global Calculator) were used as teaching tool and didactic material at Imperial College and IFP School are now described. The study was carried out once a year and for three consecutive years (2019, 2020, and 2021), during the second half of respective academic years in Europe: 2018–2019, 2019–2020, and 2020–2021. Each assessed programme has one year of duration, i.e. every year there was a different student cohort.

#### Imperial college London

The assessed programme was the *Master of Research (M.Res.) in Green Chemistry, Energy and the Environment*, which is offered by the Department of Chemistry at the Imperial’s Faculty of Natural Sciences. The programme had 23, 21 and 21 students, respectively in 2019, 2020 and 2021, from different nations every year, including the UK, China, European and Latin American nations. The age range is about 21–30 years old, as some students start the course after a 3-year BSc degree. Most students were Chemists. The lecture was carried out in the Imperial College White City Campus in London, in May 2019, whereas in May 2020 and May 2021 the lectures were online (via an asynchronous recorded lecture, followed by a synchronous activity using *MS Teams*), due to the coronavirus pandemic. The classroom had a projector and internet available. Further information about this master’s programme is available online.^9^

#### IFP school

The two assessed programmes were the *Professional Master in Energy & Markets (ENM),* and the *Master of Science (M.Sc.) in Petroleum Economics and Management (PEM)*. IFP School is the teaching unit of IFP Energies Nouvelles (formerly French Institute of Petroleum), which also includes several research laboratories in oil, gas and renewable energies, as well as an industrial training unit. Both masters here assessed are offered by the IFP School’s Centre for Energy Economics and Management (CEEM) in Rueil Malmaison Campus (greater Paris), France. Most of the ENM students were French (approximately between 22 and 25 years old), all of them were Engineers, and some of them were already working on energy companies. In contrast, the PEM had students from a more diversified background, such as Economists, Engineers and Geologists, and their ages had a slightly larger variation, approximately between 22 and 35 years old. Moreover, the PEM had students from several nations, such as France, United States, Colombia, Brazil, as well as several African and Asian countries, especially Arabic nations. Classrooms are equipped with internet and projector. IFP Energies Nouvelles is linked to the French Ministry of Industry. Further descriptions about these two master’s programmes are also available online.^10^^,^ 11 The lectures occurred in person in May 2019 and online (due to the pandemic) in May 2020 through a synchronous lecture via *Zoom* for both programmes (PEM and ENM). In May 2021, specifically, the lecture occurred for the PEM (again via distance mode) but not for the ENM. Regarding the total number of students in each programme per year, PEM had 39, 35 and 35 students in 2019, 2020 and 2021, whereas ENM had 36 and 44 students in 2019 and 2020, respectively.

### Assessment of the calculators in classroom

The first author of this article was responsible for lecturing in these three master’s programmes, as he uses to do at both Imperial College and IFP School annually for several years. Therefore, the author was already familiar with the use of system dynamics tools in classroom, given that he was one of the modellers of the Global Calculator and contributed with the European Calculator as well. However, for teachers not yet familiar with these tools, it is recommended to study and test them in advance, by using their training tutorials and guiding documents available in their respective websites (Appendix 1), in order to avoid delays and improvisation during the lecture.

After each respective lecture in 2019, 2020 and 2021, a link for a survey (questionnaire) was sent to the students to evaluate the use of system dynamics as an educational tool. The full survey is also available in Appendix 2. It starts with a brief introductory statement for contextualising its aim for the participants and for clarifying that they would not have to identify themselves, as also explained by the teacher in advance, during the lecture. This statement and explanation were made to ensure that ethics clearance was granted by the participants and to inform them that their personal identities would not be collected nor exposed as part of this study. Moreover, answering to the survey was a voluntary act and it was not part of a course evaluation nor an exam. All answers were reported individually but anonymously in line with the Chatham House Rule.^12^ The respective course coordinators were also aware of this survey and its purpose.

The questionnaire was comprised of eight questions, including questions of multiple choice and descriptive writing for evaluating the student’s individual perspectives more deeply (Lederman et al., 2002). The survey was aimed to assess the usefulness of the calculator as a teaching tool. It was not aimed at evaluating the teacher’s performance or the course more broadly, and neither at comparing the two institutions involved. The survey was prepared using *SurveyMonkey*, an online survey software. Only the students who attended the lectures received the survey’s weblink. The questionnaire was the same for all the students and assessed years.

The lectures carried out in the postgraduate programmes already described had different lengths, intensities, and student backgrounds, suggesting different impacts on learning, according to the number of hours dedicated to understanding the models. The lecture at Imperial College, for instance, was a half-day lecture, involving a collective work afterwards, in which the students were split into small groups (about five people each), being responsible for assessing a different national calculator attributed to each team. The groups then presented their respective assessments using posters (A0 format) for the different selected calculators in front of their peers few weeks later. It was an interactive activity and the respective group presentations were marked by invited academic staff, including the lecturer. The course coordinator awarded a symbolic prize to the best group presentation ever year. As for the lectures at IFP School, they were restricted to the lecturing time in classroom, although they were also interactive. For the ENM, it was a half-day lecture, whereas for the PEM, it was a full-day lecture.

In all the assessed programmes the lecturer firstly presented an overview on renewables and land-use dynamics, and their relationship with GHG emissions. It was an interactive lecture with several discussions during its explanatory part. After this, the teacher briefly explained ‘system dynamics’ and how the calculator models operate, followed by a participative use of the Global Calculator in classroom and a glimpse of national calculators.

The survey’s results were then analysed in aggregate (as percentages) and disaggregate (per postgraduate programme). In the disaggregated analysis, the results with more than 50% representation were called the “dominant group” in their respective figures. The dominant group may involve just the highest response option (if already achieving more than half of the total options) or the first and second highest response options combined and so forth, until achieving more than 50% share, showing the preferred response by most students.

### Technology and assessments

Regarding the educational *technologies* used in this research, they were all active and a blending of inquiry-based learning and project-based learning. From the point of view of the inquiry-based learning (Carvalho, 2017), we can say that the approach had an experimental learning, the didactic material was intriguing enough to grasp the student’s attention and allowed the students to manipulate it freely to obtain their own conclusions. The fact that the calculator allows the students to greatly vary their actions and view the corresponding results instantaneously, structuring their emerging regularities was a major element of engagement and learning. The lecture’s slides (including hyperlinks to the calculators and other complementary materials) used in the presentation were all sent to the students, who were encouraged to read and study them either before or after the lecturing time, similar to a *flipped classroom* approach, working as another active educational *technology*, in which the leaners study at home and take the class to share ideas and clarify any queries (Oliveira et al., 2016; Soares et al., 2019).

There was, according to what the inquiry-based educational technology proposes (Carvalho, 2017), a moment to distribute and describe the experimental material (the 2050 Calculators) and a moment for the students to run the tool interactively. At Imperial College in particular the students also had the chance to find their solutions within their groups and see each other’s solutions to make a systematic assessment of the subject. The project-based learning helped improve the collaboration within groups and, at the end of the lectures, in the case of Imperial, between the groups themselves.

In terms of summative assessment, IFP School has final written exams, which can include questions related to this lecture’s topic (among other questions from different lectures). The programme at Imperial, in contrast, marks the students (regarding this specific lecture) through their poster presentations, involving three academic staff as a panel session, with the other students also remaining in the classroom and participating in the debates.

It is important to note that the use of system dynamics in education should be included in the course syllabus, when aimed to be used as an educational tool more substantially. Moreover, systems models can be used to integrate topics from different courses, serving as an interdisciplinary platform for critical thinking and social-interactionist learning, providing major educational outputs, such as a system view of complex subjects.

## Results and discussion

The results on the use of system dynamics (calculators) for energy and environmental education are presented in two parts. The first is a brief assessment carried out about the calculators currently available worldwide, in terms of their accessibility and suitability as educational tools. The second is about the students’ perspectives on the calculators as a didactic material.

### Assessment of the 2050 Calculators as a didactic material

The main results of the assessed calculators are consolidated in Table 1 (Appendix 1), showing for each of them their main characteristics, such as if the model is operational and available online, if there is any supporting documentation available, and if the modelling algorithm (calculations) is available in open access. It also includes information about when the respective calculators were launched, their language versions, brief comments, and respective websites. The calculators are fully available in public domain, with easy access and in the languages of their respective countries (sometimes also in several languages, including English).

**Table 1:** Review on the 2050 Calculators currently available worldwide

	Webtool available	Supporting info	Calculations	Launching year	Languages	Comment	Website
Australia	Yes	Not found	Not found	2015	English	The calculator is fully operational	http://www.2050pathways.net.au/calculator
Austria	Yes	Available	Not found	2015	German	The calculator has both a light and full version, but only the light version is currently available online	http://light.klimarechner.at Currently offline: http://2050-calculator-tool.ad2book.com/
Bangladesh	No	Available	Not found	2015	English	The calculator is fully operational	http://calc.bd2050.org/
Belgium	Yes	Available	Partially available	2011 (initially only for Wallonia)	French, English, Flemish	Light and full versions are both available. See also the article on the Belgium Calculator by Berger et al. (2020)	http://pathways-calculator.be https://climat.be/2050-en/my2050-tool
Brazil	Yes	Not found	Available	2016 (calculator), 2021 (simulator)	Portuguese (the simulator is also available in English version)	Focused on energy planning. Developed by the Brazilian Energy Research Company (EPE) in collaboration with the Federal University of Rio de Janeiro (UFRJ). In 2021, UFRJ, the World Resources Institute (WRI), Energy Innovation LLC, and the Brazilian Ministry of Science, Technology, and Innovation (MCTI) launched the 2050 National Simulator for Sectorial Policies and Emissions	2050 Calculator (currently offline): http://calculadora2050brasil.epe.gov.br/calculadora.html 2050 Simulator: https://brazil.energypolicy.solutions/
China	Yes	Not found	Not found	2012	Chinese, English	The link for the webtool available on the provided website is broken. The calculator was developed by the Energy Research Institute of the China’s National Development and Reform Commission (NDRC)	https://openei.org/wiki/China_2050_Pathways_Calculator
Colombia	Yes	Available	Available	2015	Spanish	There is a game and a full version of the model	http://calculadora2050.minambiente.gov.co/
Czech Rep	Yes	Available	Available	2016	Czech	Full version of the model and all links working properly	http://co2.enviros.cz/
Ecuador	No	Not found	Not found	2016	Spanish	Developed by the former National Institute for Energy Efficiency and Renewable Energy (INER), currently Institute of Geological and Energy Research (IIGE). Apparently, the webtool is apparently no longer available	Currently offline http://plataforma.iner.ec/
India	Yes	Available	Available	2014	English	Full version of the model with all links working properly. Pathways by 2047	http://iess2047.gov.in/pathways
Indonesia	Yes	Available	Available	2014	Bahasa Indonesia	It is a complex and very well documented Calculator. There is also a Papua 2050 calculator. However, their links are both currently broken	Currently offline: http://calculator2050.esdm.go.id/ https://papua2050.wwf.id/
Ireland	Yes	Available	Available	2016	English	Very well-designed website with a variety of historical and technical documentation. It has tutorial videos and includes a light version	http://ireland2050.ie/irish-2050-calculator/ http://ireland2050.ie/my2050/
Japan	Yes	Available	Available	2014	Japanese, English	Full version available. It is called Japan 2050 Low Carbon Navigator. This navigator was assessed by Moinuddin and Kuriyama (2019)	http://www.en-2050-low-carbon-navi.jp/
Mauritius	Yes	Available	Available	2015	English	Full version available, links working properly	http://mauritius2050calculator.govmu.org/
Mexico	Yes	Available	Available	2015	Spanish	The calculator website is currently inaccessible. It includes a light version. There is also an independent assessment about the Mexico 2050 Calculator in Elizondo et al. (2017)	Currently offline: http://www.calculadoramexico2050.org/ http://mi2050.calculadoramexico2050.org/ See also: https://centromariomolina.org/calculadora-mexico-2050/
New Zealand	Yes	Available	Available	2016	English	This calculator is focused on national and local (Wellington City) approaches	http://www.climatecalculator.org.nz
Nigeria	Yes	Not found	Available	2015	English	Classical 2050 calculator	http://necal2050.energy.gov.ng/
South Africa	Yes	Available	Available	2014	English	This calculator has both a full and a light version, as well as a blog with further explanations. Note: the full version takes significant time for displaying and processing	http://sa2050pathways.environment.gov.za/ http://my2050.environment.gov.za/ https://sa2050pathwayscalculator.wordpress.com/
Southeast Europe	No	Available	Available	2015	English, Bosnian, Croatian, Albanian, Montenegrin, Macedonian	Available in several languages of Southeast Europe, in addition to English. The calculator includes an interactive game. Links currently broken	Currently offline: https://www.see2050carboncalculator.net/ https://www.see2050energymodel.net/ See also: https://seechangenetwork.org/
South Korea	No	Available	Available	2013	Korean	It includes the full version of the calculator, links working properly. It is only available in Korean language	http://2050.sejong.ac.kr/
Switzerland	Yes	Available	Available	2015	English, French, German, Italian	Full version available. It has a guide and tutorial video. Link currently broken	Currently offline: http://www.energyscope.ch/#
Taiwan	Yes	Not found	Not found	2013	English, Taiwanese	Classical version of the 2050 calculator. It includes the option to save the generated graphs as images and a light version. Websites currently broken	https://www.gov.uk/government/news/taiwan-2050-calculator-launched Currently offline: http://2050.twenergy.org.tw/ http://my2050.twenergy.org.tw/
Thailand	No	Not found	Not found	2014	Thai	It is also known as “Thai 2050 Energy Pathways Calculator” and has a light version. Apparently, the webtool is currently offline. An updated version is in progress	http://122.155.202.232/ Currently offline: http://my2050.twenergy.org.tw/
United Kingdom	Yes	Available	Available	2010	English	The UK was the pioneer in preparing a classic version of the calculator. An update version is also available, called Mackay Carbon Calculator, as well as a light version (My 2050). In addition, it has pathways by 2100	Mackay: https://mackaycarboncalculator.beis.gov.uk My 2050: https://my2050.beis.gov.uk Previous versions: Classic: http://classic.2050.org.uk 2050 Energy Calc: http://2050-calculator-tool.decc.gov.uk/ Guidance: https://www.gov.uk/guidance/2050-pathways-analysis https://www.gov.uk/guidance/carbon-calculator
United States	Yes	Yes	Not found	2021	English	Developed by the U.S. Department of Energy’s (DOE) Gateway for Accelerated Innovation in Nuclear (GAIN) through the Oak Ridge National Laboratory (ORNL), Idaho National Laboratory (INL), and Argonne National Laboratory (ANL)	https://gain.ornl.gov/#/calculator/calculate See also: https://gain.ornl.gov
Vietnam	Yes	Available	Partial Available	2015	Vietnamese	Full version. Links working properly	http://vietnamcalculator2050.atmt.gov.vn/ See also: http://www.atmt.gov.vn/default.aspx?page=news&do=list&category_id=6
European Calculator (EUCalc)	Yes	Available	Available	2020	English	Full version available. The EUCalc was led by PIK-Potsdam (Germany), involving several other European institutions, supported by the EU Horizon 2020 Programme	http://www.european-calculator.eu/ http://tool.european-calculator.eu
The Global Calculator	Yes	Available	Available	2015	Bahasa, Indonesia, English, French, Japanese, Chinese, Portuguese, Spanish	Full version available. The calculator was led by former UK DECC (currently BEIS), including several international partners, such as Imperial College London, Climact (Belgium), E&Y (India), ERI (China), WRI (EUA)	http://www.globalcalculator.org/ http://tool.globalcalculator.org

Last access of the websites cited within the table: Oct 2021. National 2050 Calculators currently in development: Kenya, Malaysia, The Philippines, and Zimbabwe. See more at: www.imperial.ac.uk/2050-calculator. See also the Climate Interactive tools at: https://old.climateinteractive.org. Source: prepared by the authors

Lecturers potentially interested in using theses calculators as a didactic material may find this list particularly useful, because it shows some basic information of all available models and includes their respective weblinks for running the webtool by themselves. Thus, the current assessment can be replicated with different students, schools, and nations. In total 27 calculators were identified, comprising 14 developing countries (52%) and 10 developed countries (37%), as well as 3 transnational tools (11%), such as the Southeast Europe Calculator, the European Calculator (EUCalc), and the Global Calculator. Therefore, the students can be encouraged not only to have critical discussions on the reality of their own nations but also to compare them with different realities worldwide, particularly in terms of international development, policy strategies, and climate change mitigation, as autonomous and critical citizens described by Freire (2005, 2011a).

### Student views on the system dynamics lecture

This section presents the main results obtained from the original questionnaire (Appendix 2) voluntarily responded by the students involved in this assessment. The following subsections show a brief discussion according to answers obtained from questionnaire, followed by some further considerations from the authors.

#### Survey’s participants

The total number of participants who voluntarily submitted their responses was 108 people, respectively 33, 41 and 34 students in 2019, 2020 and 2021, from all programmes combined. Most respondents were from the Imperial College’s M.Res. in Green Chemistry (45.4%), followed by the IFP School’s PEM (35.2%) and ENM (19.4%), which was not accessed in 2021 and, hence, had a lower share in total.

Although not all students responded to the survey, there was a representative sample of each programme’s cohorts, every year. In 2019, the response rate was 83% (19 students out of 23) for the M.Res. in Green Chemistry, 21% (8 students out of 39) for the PEM, and 17% (6 students out of 36) for the ENM. In 2020, the response rate was 71% (15 students out of 21) for the M.Res. in Green Chemistry, 31% (11 students out of 35) for the PEM, and 34% (15 students out of 44) for the ENM. Finally, in 2021, the response rate was again 71% (15 students out of 21) for the M.Res. in Green Chemistry, but 54% (19 students out of 35) for the PEM.

#### Familiarity with system dynamics models before the lecture

**Fig. 2:**
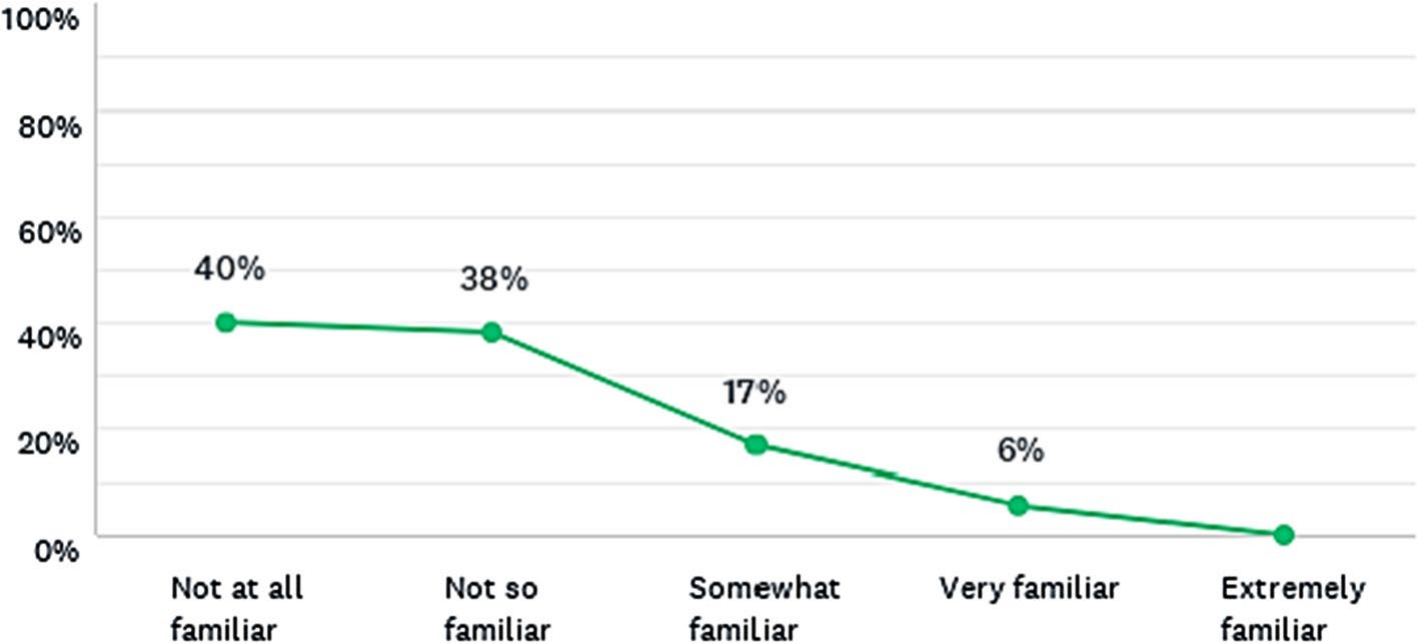
Were you already familiar with any system dynamics models, such as the calculators, before this lecture? Responses from all master’s degree programmes combined

The results show that most students had little or no knowledge of system dynamics models before the lecture, as shown in Fig. 2. None of them considered themselves as extremely familiar with this type of model. Considering that Imperial College and IFP School are both world leading institutions in science and technology, these suggests that the use of system dynamics models for environmental education on average institutions worldwide is likely to be much less common. Moreover, the participants were from postgraduate programmes and, therefore, the level of familiarity with system dynamics models in undergraduate course and secondary schools, especially in precarious institutions, is probably very rare or absent.

**Fig. 3:**
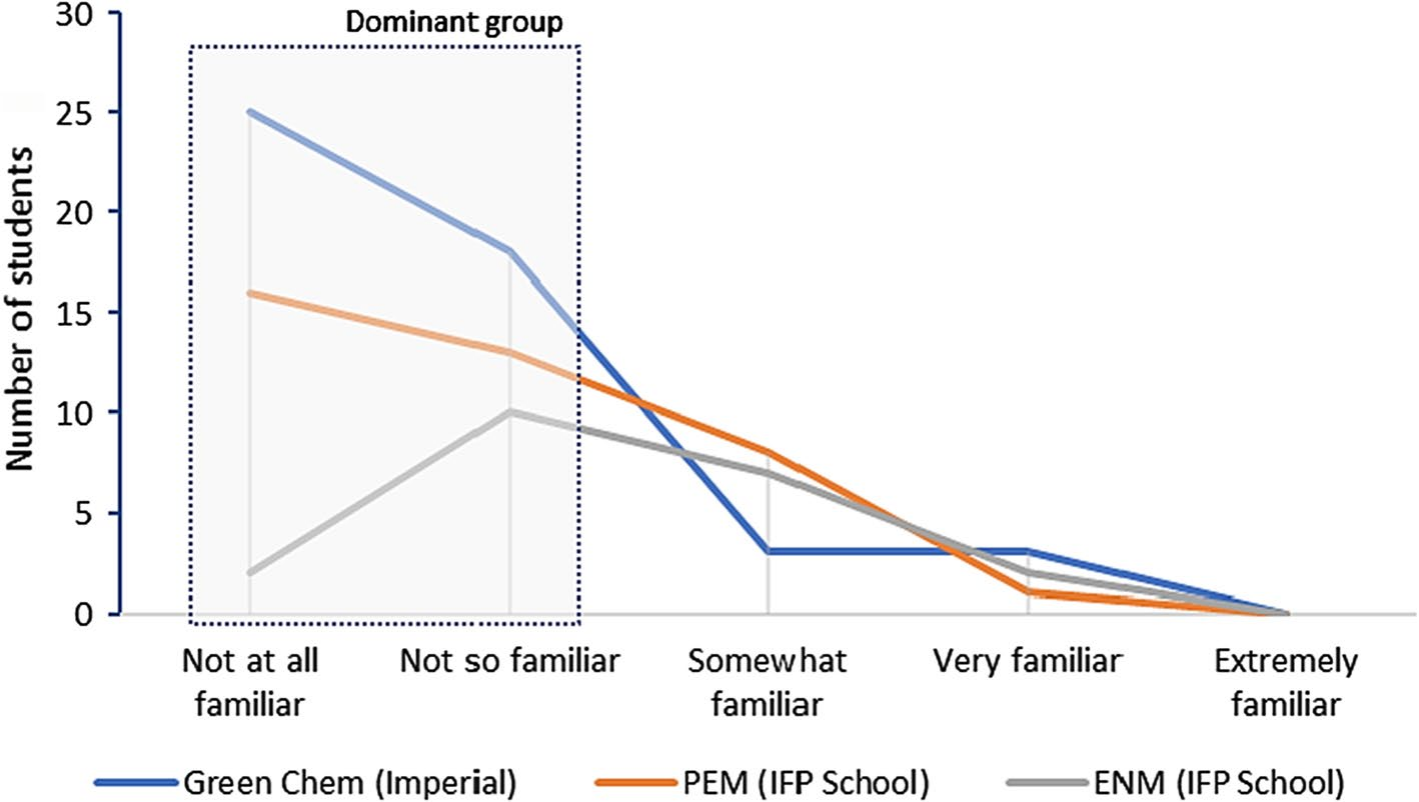
Were you already familiar with any system dynamics models, such as the calculators, before this lecture? Disaggregated responses per master’s degree programme

Moreover, the reduced familiarity with system dynamics models were relatively similar across the three master’s programmes, showing no substantial differences in the results (Fig. 3). All students responded to this question. Although some variations were observed, including due to the intrinsic subjectivity of this question, most students positioned themselves as someone below a mid-level of familiarity with the calculators, here characterised as the “dominant group”. This shows that system dynamics models were relatively new (or totally new) to most of them, not to mention their use in science education. Therefore, it is recommended that the teacher provides the students with preliminary readings or introductory videos before the lecturing time (e.g. about a week in advance) and stimulates discussions in classroom, whilst also clarifying and introducing key concepts to them, like a “flipped-classroom” model (Oliveira et al., 2016; Soares et al., 2019). The teacher can also encourage group discussions, preferably joining those more familiar with system dynamics with those not familiar at all in a same group so that they can learn with each other, rather than leaving them for a random distribution, which may result in unbalanced groups. In the case of using National 2050 Calculators in an international classroom, the students could be stimulated to be organised in teams with members from different nationalities and to assess collectively a calculator that none of them is originally from in order to stimulate them to think in different perspectives.

#### Learning through system dynamics models

**Fig. 4:**
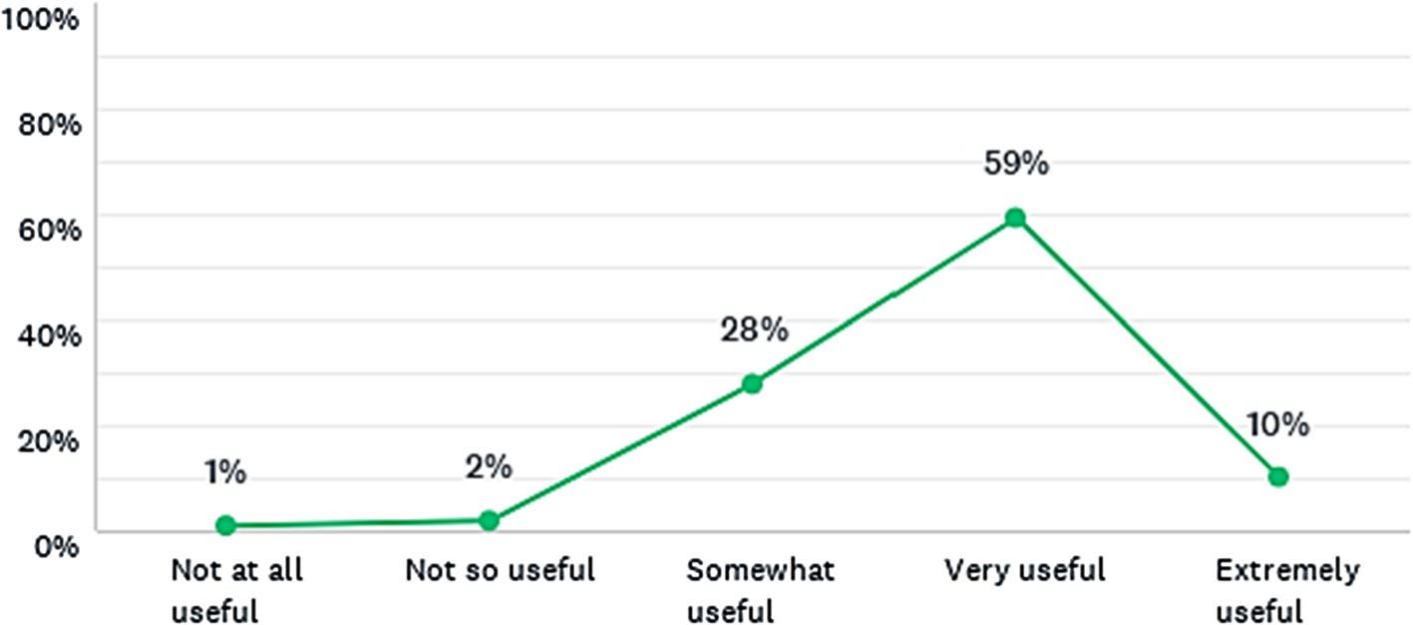
Is it useful for learning to run system dynamics models in classroom, such as the calculators? Responses from all master’s degree programmes combined

The calculators were found to be useful tools for learning complex environmental issues, such as climate change, energy, and land use. Only a few students disregarded them as useful for learning (Fig. 4) and all participants responded to this question. While running these models with the master students, an increase in the level of interaction among the students and between the students and the teacher, compared to the part of the lecture without using the tool was noticed. Therefore, the models appear to facilitate the process of “active learning” in classrooms from the perspective of inquiry-based learning.

**Fig. 5:**
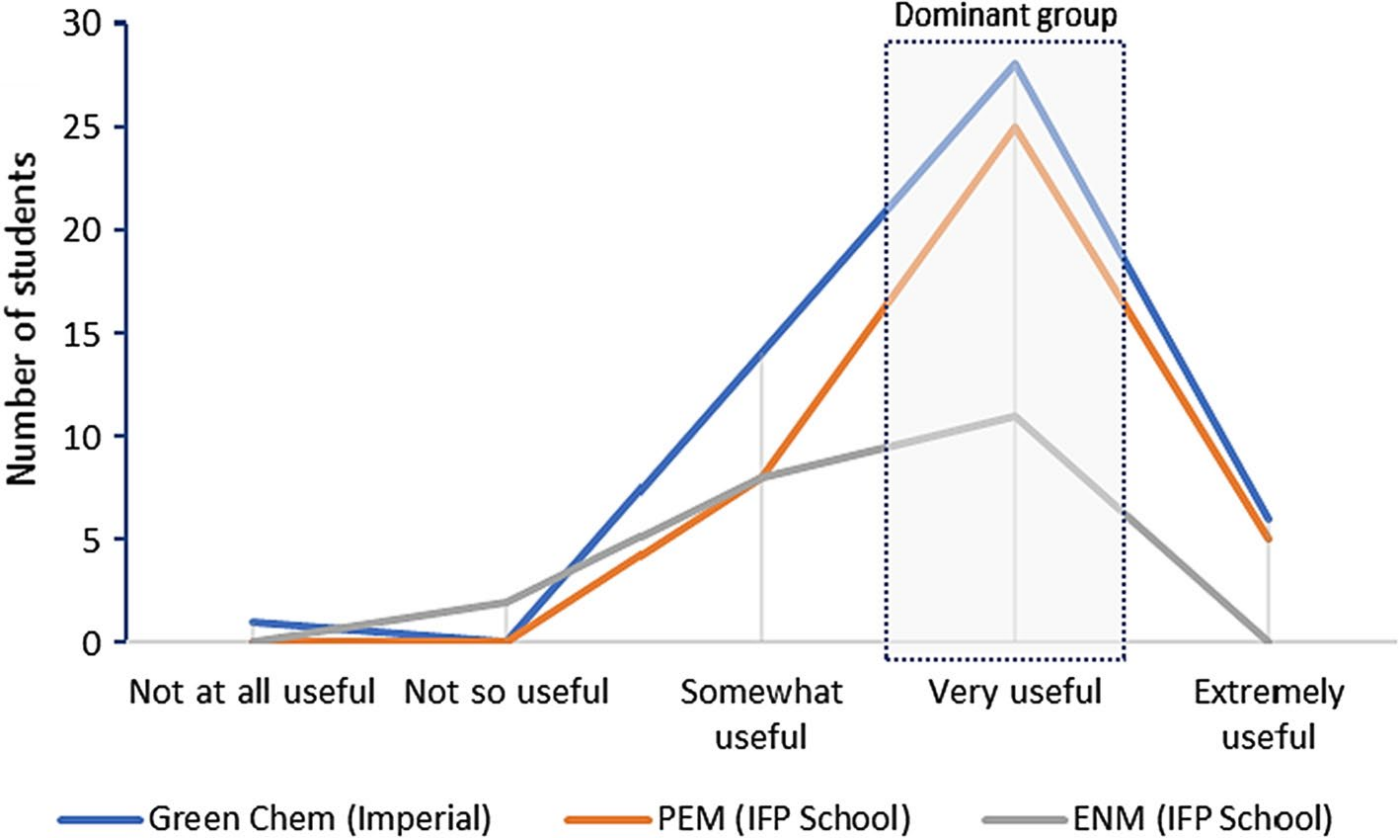
Is it useful for learning to run system dynamics models in classroom, such as the calculators? Disaggregated responses per master’s degree programme

The results split into the different postgraduate programmes show some variation, but all students considered that the use of system dynamics for learning was at least somewhat useful, whereas the vast majority considered it very useful (Fig. 5). To find something useful or not depends on several aspects such as the students’ professional experiences and future perspectives, as well as their background and personal interests, let alone the lecturer’s performance, which may change from one programme to the other, potentially affecting the results.

The proposed new knowledge needs to mean something for the student to find it useful, i.e. it must dialogue with the student’s reality (Freire, 2005). In this context, assessing the students’ profile and the main characteristics of each programme may help identify the possible reasons for the variations obtained in the results for this question. As briefly mentioned, the ENM programme, for example, is a professional masters in energy and markets aimed at engineers, in which many students were already working in the energy sector, especially in energy companies based in France (e.g. oil and gas, and electricity businesses). Thus, apparently, some students might be more concerned with the practicality of the professional use of system dynamics approaches like the calculator either in their companies or for market analysis, which, in fact, is not the focus of the 2050 Calculators. In addition, the ENM had the class with the least number of hours dedicated for running the system dynamics models and understanding the calculators more deeply and, thus, a larger number of hours could potentially affect this result towards a similar pattern to the other two assessed programmes.

As for the PEM, most students considered the 2050 calculator very useful. The students had varied types of academic background, from human sciences to engineering degrees, and only a few of them were simultaneously working in the energy sector whilst doing their studies. Students were coming from several nations, which contributed for intensive international discussions during the lecture, potentially impacting their very positive perception on the usefulness of system dynamics models for learning.

Regarding Imperial College, most students also found that running system dynamics models in classroom was very useful for learning. Many students were clearly interested in climate change affairs in their master studies, some of them already considered applying for a doctorate degree afterwards. Most students were only studying whilst taking their masters and the group was very international. Moreover, it was the programme in which the students were able to interact more with the calculators, including extra-class activities, such as the poster’s presentation already described. It was also reported that the learners have enjoyed the lecture using the calculators, because they offered them a different perspective on energy policy and climate change mitigation strategies, compared to other courses in their regular programme, which is more focused on green chemistry. All these aspects may help explain (at least partially) their positive perceptions.

#### Understanding the calculator’s logic in classroom

**Fig. 6:**
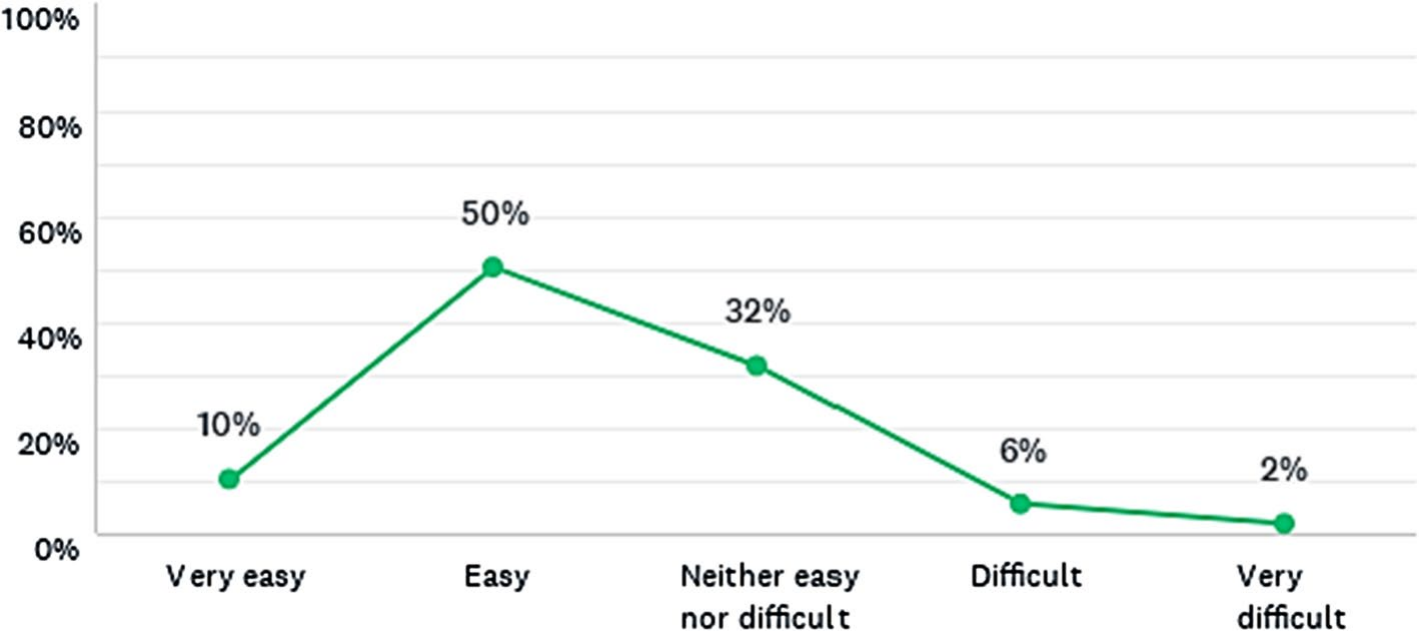
Was it easy to understand the calculator’s logic in classroom? Responses from all master’s degree programmes combined

As shown in Fig. 6, the calculators presented some level of challenge to be understood, given that most students found it easy or neither easy nor difficult to understand,^13^ although some students considered understanding the calculators either as something very easy or difficult/very difficult. Thus, in general, the results suggest that the lecture occurred within the limits of the students’ *zone of proximal development*, i.e. within the distance between the actual development level and the potential development level of each student, as proposed by Vygotsky (1991, 2000). According to him, the learning process can be effective when it occurs within this zone.

Hence, it is important to ensure that the students have the basic knowledge to understand the main dynamics involved in the models, avoiding any student finding it too difficult. Ausubel (Moreira, 2011) calls this previous knowledge as *advance organisers*, which are necessary for a meaningful learning as part of the students’ *cognitive structures* (Ferreira et al., 2020; Silva Filho & Ferreira, 2018; Silva Filho et al., 2021). These *cognitive structures* consist of a hierarchical structure of concepts, which represent the sensorial experiences of each person (Moreira, 2011).

Therefore, the knowledge organisation is fundamental for a meaningful learning, as well as the role of the teacher as a mediator. For Vygotsky, this mediation involves the use of instruments (e.g. a tool) and signs (e.g. icon, index, symbol) (Moreira, 2011). This thought also evokes the *theory of mental models* proposed by Johnson-Laird (1995, 2010). His theory involves mental representations (i.e. an internal process) and external representations (i.e. pictorial and linguistics), whilst also making some analogies with computing processes (Moreira, 2011). These concepts from both Vygotsky and Johnson-Laird are important to better understand the learning of system dynamics, given that using the calculators requires abstraction, whilst assessing icons, indices, textual and mathematical symbols, and graphical representations. Thus, if the student is not attempted and motivated to learn the calculators or does not have the basic knowledge to understand the main associated concepts, the models may be quite confusing at first, apart from their user-friendly interfaces.

**Fig. 7:**
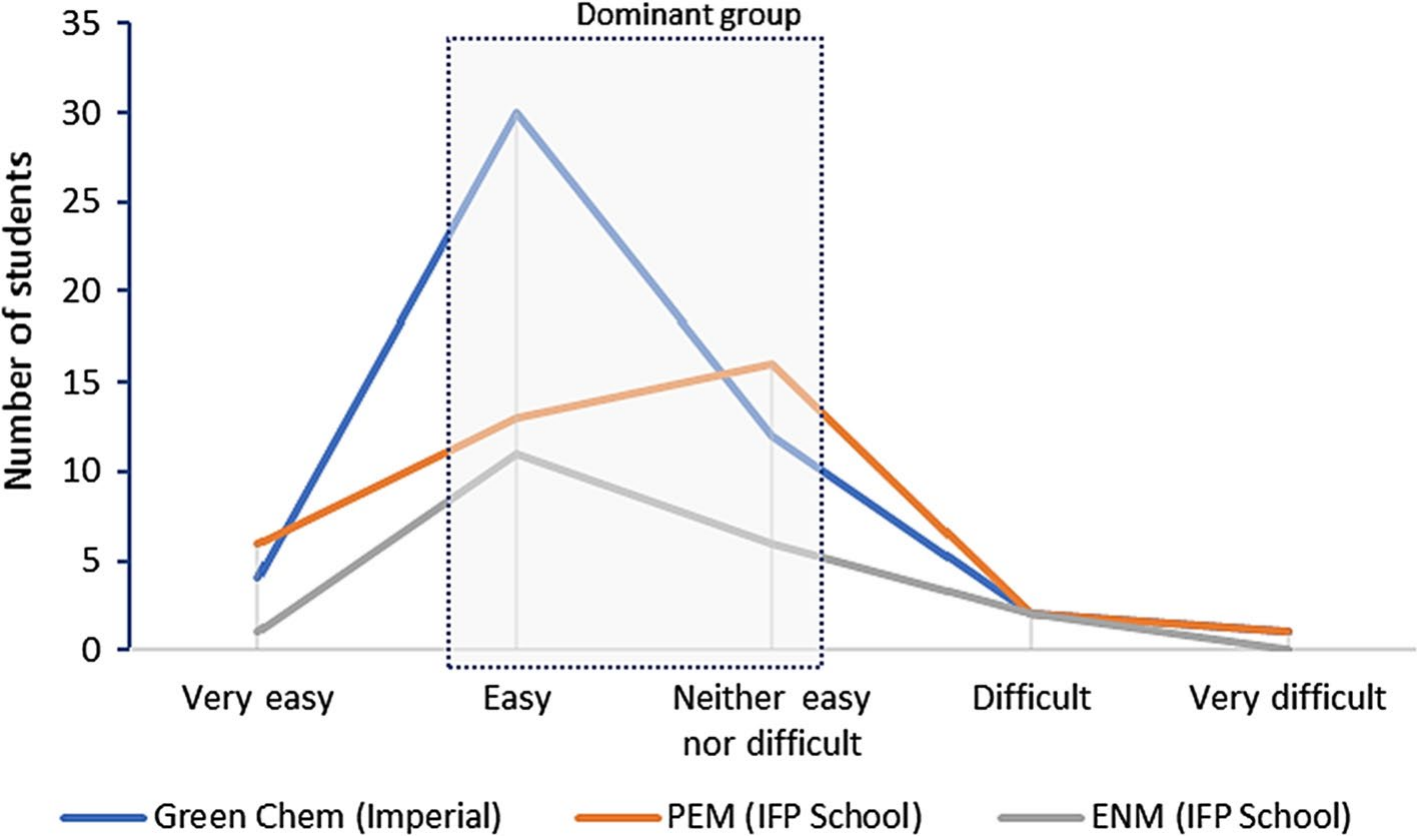
Was it easy to understand the calculator’s logic in classroom? Disaggregated responses per master’s degree programme

The disaggregated results per type of postgraduate programme show some variations, but most students found the calculators’ logic (rationale) within a moderate level of difficulty to understand (Fig. 7), rather than in the extremes of very easy and very difficult. The dominant group in general found it relatively easy, but in the case of PEM specifically, the majority had a peak in “neither easy nor difficult”. Only one student skipped to answer to this question. Furthermore, two students from different programmes found it very difficult, which suggests that a further attention must be given to these specific learners in order to identify the main difficulties involved and reasons behind them. This could also help the teacher to provide a more focused didactic strategy to assist these students overcome their difficulties more easily, rather than simply ignoring this variation. As discussed by Freire (2011a), teaching requires listening, dialogue, and generosity. According to Vygotsky’s *learning pathways* (Vadeboncoeur & Collie, 2013), the teacher must respect the limitations of each person. Students have different contexts and personal histories and, therefore, the teacher must have enough alterity in order not to leave any student behind (Habowski et al., 2018).

On the other hand, it is not always possible for the educator to reach all students efficiently, particularly when it is a very diversified class and with too many students; however, the teacher must pursue a greater equality. For instance, whilst running the calculators together with the students inside a classroom, it is important to adjust the lecture according to the students’ background as well as to present and contextualise the main knowledge necessary to understand the tools. This should be done preferably in the first part of the lecture, or in a previous lecture.

#### On the frequency of using systems models in science education

It was unanimous among the participants that systems models should be used more often for science education and strategic thinking. There was also a choice to argue textually, rather than yes or no, but none used this option. This positive feedback suggests that system dynamics models such as the 2050 Calculators were considered appropriate for their learning process. The list of 2050 Calculators described in Appendix 1 may help disseminate these initiatives.

#### Reflections on policy making supported by the 2050 calculators

**Fig. 8:**
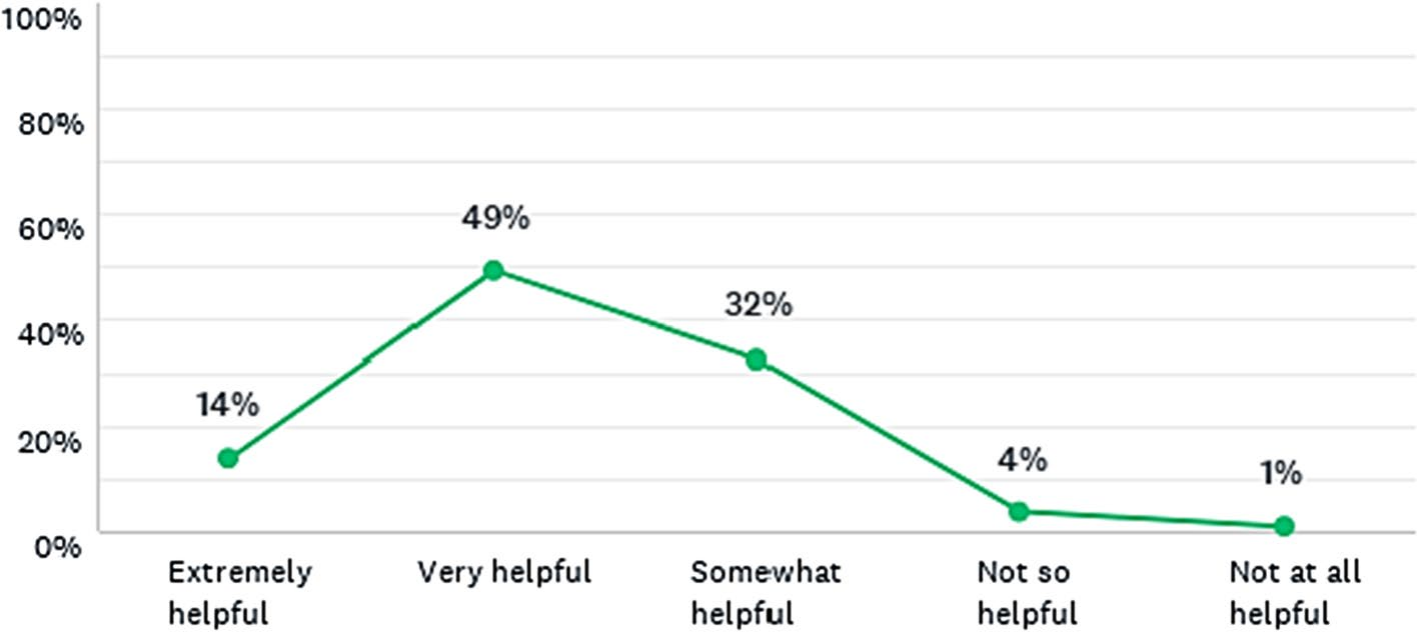
Do you think that the discussions made in classroom, supported by the calculator, were helpful to have any type of reflection on policy making? Responses from all master’s degree programmes combined

As shown in Fig. 8, most students found the discussions based on the 2050 Calculator very helpful, somewhat helpful, or extremely helpful, although a few students considered it not so helpful or not at all helpful to have reflections on policy making. Therefore, the calculators may also contribute towards a critical thinking and political awareness, which aligns with the Freire’s (2005, 2011a, 2011b) concept of critical pedagogy, in which education should be a vehicle of social transformation and not only a mere obtention of transferred knowledge.

**Fig. 9:**
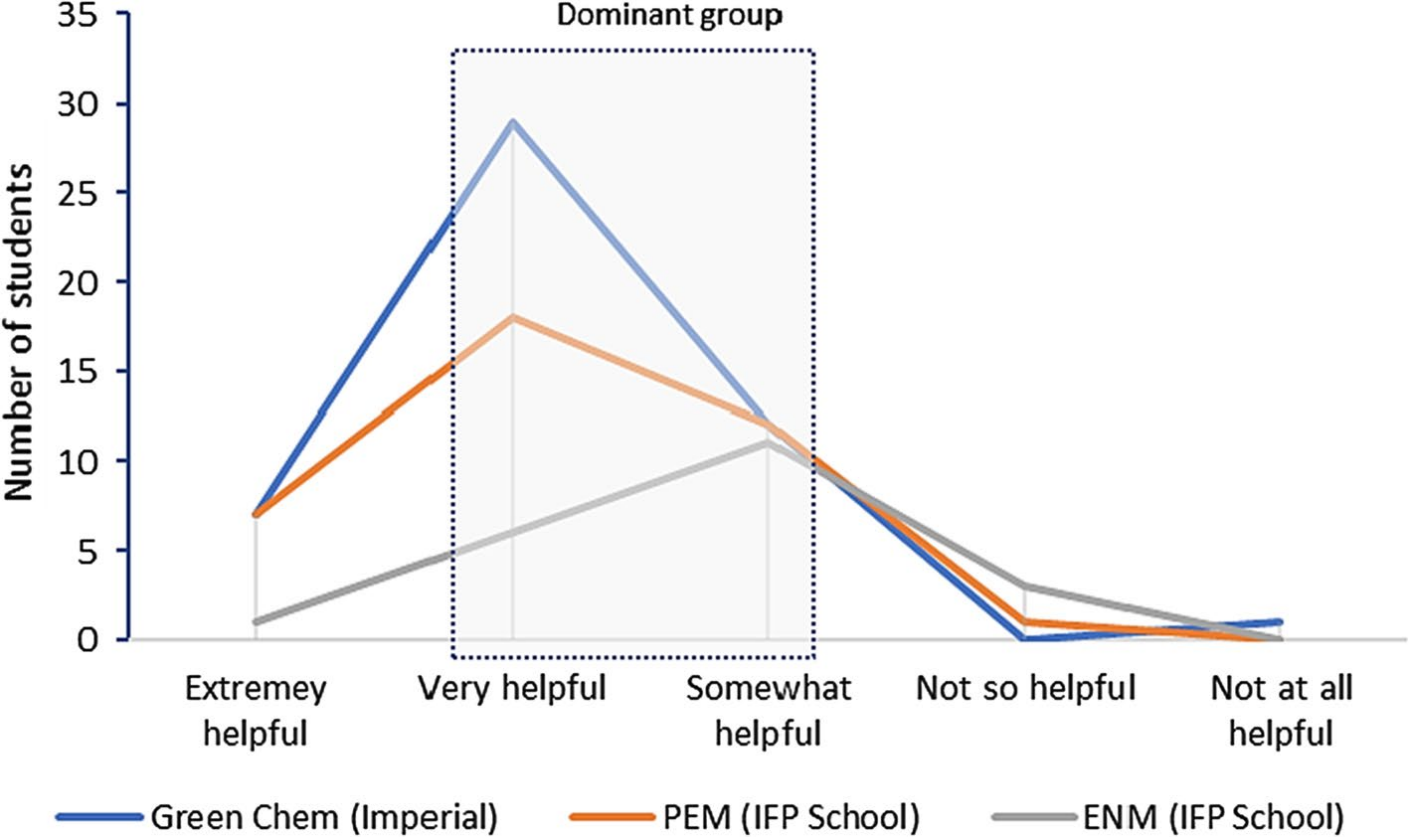
Do you think that the discussions made in classroom, supported by the calculator, were helpful to have any type of reflection on policy making? Disaggregated responses per master’s programme

The disaggregated results show some variations across the three master’s degree programmes, but the majority of learners considered the 2050 calculators helpful (Fig. 9). This result is especially important, because the 2050 Calculators are aimed at informing the climate debate, which requires urgent action. The international climate debate is highly controversial, with some global leaders and policy makers still neglecting it, apart from the substantial scientific evidence already available (Holdren, 2021; IPCC, 2013). In the context of the Paulo Freire’s Pedagogy of the Autonomy (Freire, 2011a), teaching requires to understand that education is a way to transform the world and to recognise that it is politically ideological. This does not mean that the teacher should influence the students with her or his personal political views, but to encourage the students to think critically about their own realities towards a broader social transformation.

Furthermore, the results shown in Fig. 9 may vary depending on the way the lecturer conducts the learning process and the amount of time for having policy discussions based on insights from the calculator (as reference tools of system dynamics, among other options). The course at Imperial had more time available for discussions, followed by the PEM and ENM. This suggests that the more time for debating, the larger the perception on its use for reflecting on policy making. Although the 2050 calculators are useful for illustrating lectures on complex topics such as climate change, their impacts on the real life of the students and their environment depend on how these models are used and discussed in classroom. Hence, the teacher’s role as a facilitator must be ethical and encourage the students to think by themselves. In this context, while demonstrating the tools, it is important to contextualise the topics according to the students’ realities in advance and during the lecture.

Moreover, it was observed by the lecturer that by combining the epistemological approach proposed by Freire (2005, 2011a) with the use of systems models (INCOSE, 2015; Voinov, 2008), it was possible to stimulate discussions about how changes in energy production and consumption could affect carbon emissions, and how changes in lifestyle (e.g. dietary patterns, use of transport) could impact on these emissions too. By using the calculators, the students were able to identify some critical issues that have not been sufficiently addressed by policy makers and business leaders yet. Thus, based on these findings, system dynamics can be used as relevant complementary approach to help students obtain both a critical knowledge and a systemic perspective on policy making, which is aligned with a broader social interest and sustainable development at local and global levels.

#### Achieving an interdisciplinary understanding of complex contemporary issues

When asked how the use of system models could contribute more effectively towards an interdisciplinary understanding of complex contemporary issues, such as climate change, most students were enthused in their responses. About 57% of the participants responded to this question, and 43% skipped it. The responses substantially varied, but some of the highlights are following summarised: (i) understanding the interactions, trade-offs, and calculations involved more deeply; (ii) having more seminars and workshops about these models; (iii) introducing these models earlier in schools to drive interest and knowledge of them from a younger age; (iv) keeping models updated; (v) combining the use of different disciplines to understand these models; (vi) assessing how changes in human behaviour can affect the climate; (vii) raising awareness and considering a wider picture rather than focusing on just one aspect of energy resources; (viii) helping governments to make more realistic goals, and the individuals and organisations to monitor them.

#### Final considerations

The students were also asked if they had any other comments or suggestions. Most of the respondents (65%) did not reply to this question, but some of them (35%) did and in detail. The main highlights are following summarised: (i) to do the modelling code with the students; (ii) to use the calculators preferably in person with discussions in classroom (a comment made by a student in distance learning, due to the covid pandemic); (iii) to have further explanations on the scenarios and costs involved; (iv) to have the calculator available for other nations, such as the United States. Regarding the comment on working on the programming code directly, this could be an interesting lecture for students interested in exploring these tools more deeply, but it would require a more in-depth series of lectures to be accomplished. As for the United States calculator, the U.S. Department of Energy (DOE) recently made available online its own calculator, as described in Appendix 1. In addition, several students commented that they liked the lecturing format and the experience of using system dynamics models.

The transferability of these findings depends on characteristics of the students, lecturers, courses, and type of model. This study was based on the use of 2050 Calculators, whereas other types of system dynamics approaches may have different impacts on learning and teaching practices. Lecturers interested in using either the calculators or other system dynamics models should test their use in advance, i.e. before adopting them as a learning tool with the students. Equally important is to contextualise their use in the respective course syllabuses and to adjust the lecture according to the students’ profiles.

### Limitations involved in the use of system dynamics for education

The use of system dynamics models such as the 2050 Calculators for energy and environment education has proved to be useful, but some limitations are involved, such as their visual approach, transparency, and complexity, as well the teacher’s skills, college infrastructure (e.g. need of a projector and internet), and the students’ background. Besides, some calculators have broken links and are not fully operational, whilst others are restricted to local languages and only the Global Calculator has some cost estimates included. While some calculators have shown a reasonable accuracy level and provided relevant documentation and introductory videos, others fell short in this regard (Appendix 1). Thus, the results obtained in this research could be more positive by improving the existing calculators and developing new systems models, especially those dedicated for science education and sustainable development strategies.

Moreover, system dynamics models also have limitations (Featherston & Doolan, 2012) and should be complemented by other modelling approaches whenever necessary, such as those aimed at decision-making process (e.g. game theory). In addition, carbon footprint calculators in general (i.e. not only 2050 Calculators) often do not include aspects related to lifestyle changes, connecting consumer’s behaviour to climate action (Burgui-Burgui & Chuvieco, 2020). The Global Calculator, the EUCalc and some national 2050 Calculators effectively include some related analysis, but several others still present major constraints in this regard.

The survey had some limitations as well. As already mentioned, some questions have an intrinsic subjectivity, given that the answers reflect the personal perception of the students about the calculators and the use of system dynamics in science education, particularly in energy and environment. The relatively small number of students involved in this assessment is also a limitation for the transferability of the results here shown. Future assessments could provide further evidence on the usefulness of system dynamics as effective learning tools.

## Conclusions

System dynamics models can contribute towards a critical education in energy and environmental sciences, among other areas, especially on systemic problems such as climate change. The current assessment demonstrated that most of the available calculators provide an accessible tool for science education, although some of them are not fully available or are limited in terms of supporting documentation and modelling accuracy. By running the calculators, it was possible to have a comprehensive view on interdisciplinary issues and develop graphical concepts using online models, whilst also obtaining a critical perspective on the complexity involved in energy and environmental dynamics.

Therefore, system dynamics can serve as a supporting didactic material for critical pedagogy. Relevant assessments were conducted on the use of system dynamics in education in the past (Forrester, 1992, 2009; Gould-Kreutzer, 1993; Warren & Langley, 1999), whereas the present study adds new contributions based on empirical evidence. Further studies are recommended in the context of new technologies available, particularly on the use of web-based system dynamics models as learning tools to help empower the society and stimulate systems thinking at educational level, including primary and secondary schools, as well as the use of different pedagogical approaches to science education.

## Note

The results and discussion presented in this paper do not necessarily represent the views of Imperial College London or IFP School and neither the views of their respective postgraduate programmes. The opinions here shown are those from the authors alone or their interpretations from the surveys.

## Data Availability

Not applicable.

## Appendix 1

See Table 1.

## Appendix 2

### Original survey:

#### The Use of System Dynamics in Science Education

##### Introduction about this survey

This survey is dedicated to the students who recently had a lecture with “*the first author of this manuscript”* from Imperial College London about bioenergy and renewable energies. This lecture included a brief demonstration on the use of a system dynamics model called the Global Calculator. Several other types of 2050 calculators for climate change mitigation are also available online, such as national, regional and city level calculators.

The objective of this questionnaire is to have your personal views and comments on the usefulness of this type of model as an educational tool. There is no need for identification. Any information will be treated anonymously. Please answer to this questionnaire only once. Thank you.

1. Are you a student from which master's degree programme below?

__Green Chemistry (Imperial College London)

__Petroleum Economics and Management (IFP School)

__Energy and Markets (IFP School)

2. Were you already familiar with any system dynamics models, such as the calculators, before this lecture?

__Not at all familiar

__Not so familiar

__Somewhat familiar

__Very familiar

__Extremely familiar

3. Is it useful for learning to run system dynamics models in classroom, such as the calculators?

__Not at all useful

__Not so useful

__Somewhat useful

__Very useful

__Extremely useful

4. Was it easy to understand the calculator’s logic in classroom?

__Very easy

__Easy

__Neither easy nor difficult

__Difficult

__Very difficult

__If you had any difficulties, please comment:

5. Do you think this type of system models should be used more often for science education and strategic thinking?

__Yes

__No

__Other (please specify):

6. Do you think that the discussions made in classroom, supported by the calculator, were helpful to have any type of reflection on policy making?

__Extremely helpful

__Very helpful

__Somewhat helpful

__ Not so helpful

__Not at all helpful

7. In your view, how the use of system models could contribute more effectively towards an interdisciplinary understanding of complex contemporary issues, such as climate change?

Answer:

8. Do you have any other comments or suggestions?

Answer:

*(end of survey)*

## Footnotes

1 The updated version of the UK 2050 Calculator is called MacKay Carbon Calculator and the webtool is available at: https://mackaycarboncalculator.beis.gov.uk [accessed Oct 2021].

2 See the Global Calculator webtool at: https://www.globalcalculator.org [accessed Oct 2021].

3 The FT Calculator is available at: https://ig.ft.com/sites/climate-change-calculator/ [accessed Oct 2021].

4 The EU Calculator is available at: http://www.european-calculator.eu/ [accessed Oct 2021].

5 See the Bulb Energy’s Calculator Footprint Calculator at: https://bulb.co.uk/carbon-calculator/ [accessed Oct 2021].

6 Video 1 (Introduction on the Global Calculator): www.youtube.com/watch?v=TbCg0fw3Ju8 [accessed Oct 2021].

7 Video 2 (how to use the Global Calculator): www.youtube.com/watch?v=2_6flonHN0o [accessed Oct 2021].

8 More information on the SDGs is available at: https://sdgs.un.org/goals [accessed Oct 2021].

9 Further information on the M.Res. in Green Chemistry at Imperial College: https://www.imperial.ac.uk/study/pg/chemistry/green-chemistry/ [accessed Oct 2021].

10 IFP School’s ENM Programme: https://www.ifp-school.com/en/training/specialized-engineering-graduate-degree/energy-and-markets [accessed Oct 2021].

11 IFP School’s PEM Programme (from 2022 called *Energy Technology Economics and Management*): https://www.ifp-school.com/formation/diplome-dingenieur-specialise-disdesa/energy-technology-economics-and-management-ex-pem [accessed Oct 2021].

12 Chatham House Rule: “When a meeting, or part thereof, is held under the Chatham House Rule, participants are free to use the information received, but neither the identity nor the affiliation of the speaker(s), nor that of any other participant, may be revealed”.

13 This question also had the option of providing textual comments, if the student had any difficulties, but only some of them used this option. In general, the comments suggested that it takes some time to understand the model and the parameters involved, implying that an extended lecturing time would be beneficial for that.
